# Respiratory quotients of particle-associated microbes track carbon flux attenuation in the mesopelagic Southern Ocean

**DOI:** 10.1093/ismejo/wraf255

**Published:** 2025-11-20

**Authors:** Fraser Kennedy, Matthieu Bressac, Philip Butterworth, Svenja Halfter, Philip W Boyd

**Affiliations:** Institute for Marine and Antarctic Studies, University of Tasmania, Hobart, TAS 7001, Australia; Institute for Marine and Antarctic Studies, University of Tasmania, Hobart, TAS 7001, Australia; CNRS, Laboratoire d’Océanographie de Villefranche, Villefranche-sur-Mer, Sorbonne Université, 69400, France; Institute for Marine and Antarctic Studies, University of Tasmania, Hobart, TAS 7001, Australia; Earth Sciences New Zealand, Wellington, 6021, New Zealand; Institute for Marine and Antarctic Studies, University of Tasmania, Hobart, TAS 7001, Australia

**Keywords:** respiration, apparent respiratory quotient, microbial, mesopelagic, biogeochemistry, particle degradation

## Abstract

Mesopelagic microbes and zooplankton, degrade, and attenuate >90% of the 10 billion tonnes of particulate organic carbon that sinks into the oceans’ interior annually. Approaches such as particle interceptors/incubators (called c-respire) can isolate the microbial assemblage attached to particles from that of zooplankton, enabling quantification of microbially mediated particulate organic carbon flux attenuation. This metric yields patterns of particulate organic carbon degradation by microorganisms through the upper mesopelagic (200–500 m depth). Here, we investigate the temporal sequence of particulate organic carbon degradation in two steps. First, we intercept sinking particle assemblages from different depths (180–300 m) and hence with varying degrees of exposure to microbial activity. Second, we incubate these intercepted particles shipboard for 12 h (short-term) and track degradation using apparent respiratory quotients (dDIC/dDO_2_). We also conducted a 12-h shipboard incubation on a particle assemblage that had already undergone a 36-h *in situ* c-respire (long-term) incubation. At a subantarctic and two polar sites, apparent respiratory quotients (ARQs) from short-term incubations exhibited a significant decrease with depth, consistent with particles deeper in the upper mesopelagic being exposed to a longer period of degradation and flux attenuation (as they settle). ARQs from all long-term incubations had significantly lower ARQs, and smaller depth-dependent gradients, than the short-term incubations. We interpret these trends as being driven in part by sequential changes in the stoichiometry of the microbially altered particulate organic carbon (POC) substrates. ARQs of <0.5 (less than the theoretical minimum) were observed in long-term incubations suggesting a role for incomplete oxidation of dissolved substrates. This temporal sequence is used to conceptually explore what sets the limits on microbially mediated degradation of POC.

## Introduction

Downward particulate organic carbon (POC) export flux has been the central metric of biological pump research for decades [1, 2]. Much of our understanding is derived from the interpretation of patterns of what remains—POC flux [3]—rather than what is degraded to dissolved constituents. Modelling studies estimate that billions of tonnes of POC exit surface waters annually [4] and ~90% is attenuated [5], transformed to dissolved organic carbon (DOC) and/or dissolved inorganic carbon (DIC) within the mesopelagic (200–1000 m). Such transformations have major implications for the carbon cycle [6], and macro-nutrient resupply [7]. To resolve how the biological pump influences atmospheric CO_2_ concentrations also requires quantification of the fate of degraded POC to DOC and/or DIC [8]. Hence, a better mechanistic understanding of how particles are transformed by zooplankton and microbes is required. The ability to separate mesopelagic particle degradation by microbes from that mediated by zooplankton, using the c-respire dual particle interceptor/incubator (Fig. S1), enables exploration of depth-related patterns in microbially mediated particle degradation [9].

Sinking particles represent a diverse inventory of biochemical substrates [10, 11], and hence are colonised by microbes [12] and/or consumed by zooplankton [13]. Such particles are a distinctive microbial niche since their community composition [14] and enzymatic activities [15] differ from that of free-living bacteria in the surrounding waters. This attached microbial community subsequently sets the metabolite composition of particles and hence their lability [16]. As particles sink through the mesopelagic, they are degraded hydrolytically mainly by exoenzymes from attached bacteria [17, 18] contributing to particle flux attenuation. Hence, the reactivity or lability of substrates associated with settling particles plays a key role in determining how much POC sinks to depth [19], e.g. by altering characteristics such as size and sinking rate [20, 21].

Insights from approaches including epifluorescence microscopy [22], and biomarkers [11] suggest that the reactivity of particle-bound substrates decreased with depth presumably due to selective removal of more readily degraded components [23–25]. Recent marine microbial models represent the lability of substrates as an ecosystem property to explore the interplay of community structure, particle reactivity and to project downward POC flux attenuation [26]. There is extensive evidence from both the marine organic geochemistry of sediments [27] and soil microbial [28] literature of biochemical sequences of particle degradation. These observed sequences in marine sediments and terrestrial soils have been used to explore the theoretical basis that underpins such patterns, resulting in thermodynamic [29] and, at finer scale detail, energetic [30], or hierarchical (i.e. joint modelling of degradation and sediment burial in aquatic systems, [31] approaches to map theoretically decreases in reactivity driven by degradation.

Many studies of marine organic matter degradation have focussed on sediments (e.g. [32, 33]), and on water column particles (e.g. [34, 35]). A useful metric to link the carbon and oxygen cycles with the degradation of water column particles is the apparent respiratory quotient (ARQ, dDIC/DO_2_) [36]. As ARQs reflect substrate quality and the interlinked degree of substrate oxidation, and/or microbial metabolic pathways, with careful interpretation they provide insights into the nature of organic matter being oxidised [36]. For example, under lab-controlled conditions, shifts in ARQ from unity to 0.7 revealed different metabolic pathways driven by oxidation of carbohydrate versus lipids, respectively [37]. However, in field studies, it is difficult to tease apart zooplankton and microbial respiration in relation to their roles in particle degradation, and so ARQs can only be linked to community respiration [38]. Hence, ARQs have mainly been employed in lab-based bacterial cultures to investigate the linkages between respiratory metabolism under different substrates [37]. Although studies have focused on the degradation of proteins [39] or lipids [40] associated with particles, the specific role of microbes in setting the sequence of degradation and associated biochemical changes on settling particles is a major gap in our understanding of the fate of sinking POC in the oceans’ interior.

**Figure 1:**
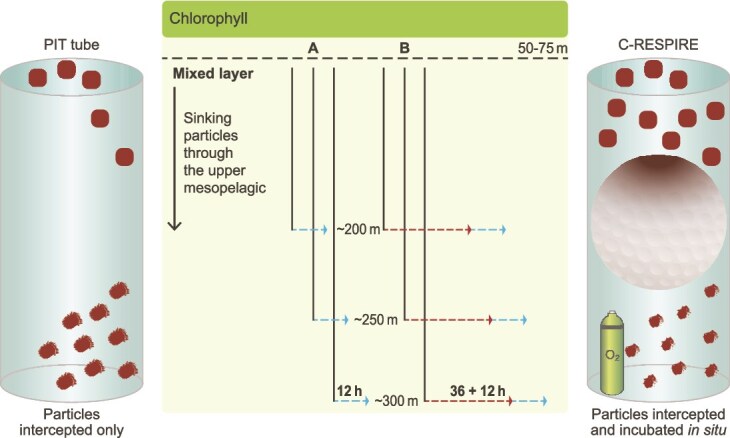
Employing depth and time to jointly explore the temporal sequence of particle degradation**.** Particles were intercepted from three depths in pit traps (left), and c-respire (right) underlying the surface mixed layer coincident with upper ocean chlorophyll (there was a DCM at PFF1 to 90 m depth). The sinking particle assemblage at each depth has likely undergone prior degradation by attached microbes and flux feeders, resulting in less labile particles at depth, as reported for the NE Atlantic [22]. Particles were intercepted at ~200, ~250, and ~300 m depth (three vertical blue lines) by pit traps for 72 h in (A), and by c-respire for 36 h in (B). In (A) subsequent short-term 12 h shipboard incubations are denoted by blue horizontal dashed lines. In (B) intercepted particles were subsequently incubated in situ (red horizontal dashed lines) for 36 h in c-respire followed by shipboard incubation for 12 h (blue horizontal dashed lines). Hence, our investigation of the sequence of microbially mediated particle degradation uses both depth (particle interception) and time (incubations). Note it is problematic to incorporate particle sinking rates to this figure as they vary with particle size from 17 m d^−1^ to 136 m d^−1^ (Table S6) based on the analysis of 14 size classes of sinking particles observed using a UVP6 particle imager on a profiling robotic float at the SOTS site (Fig. 2A) deployed on a multiyear mission during the SOLACE voyage [45].

Here, we use the ability of the c-respire particle interceptor/incubator to isolate particle-attached microbes *in situ* [41], along with lab-based incubations of previously intercepted particles to explore microbial POC degradation. This enables assessment of temporal and vertical patterns in microbially mediated degradation of the sinking particle assemblage in the upper mesopelagic. A combination of sampling the particle assemblage at different depths (and hence different exposure times to particle transformations) along with shipboard and *in situ* particle incubations was employed (Fig. 1). Concurrent measurements of CO_2_ production (converted to DIC) and oxygen consumption [37] tracked how ARQs change with depth as a proxy for particle degradation. Trends in ARQ are interpreted using insights from marine biochemistry [42] along with lab experiments focusing on particle types including zooplankton fecal pellets [43], marine snow [17], or using ARQ time-series [37] to conceptually explore the sequence of particle degradation. Such a model will improve the design of future field and lab studies needed to provide the requisite detail for numerical modellers [26, 44], moving beyond the limitations of downward POC flux observations to better understand the functioning of the mesopelagic C cycle. To understand the fate of 9 billion tonnes of POC flux attenuation annually in the mesopelagic [5], a key aim is to elucidate what sets the limit on how much POC flux can be broken down by microbes in this zone.

## Materials and methods

### Sites

**Figure 2:**
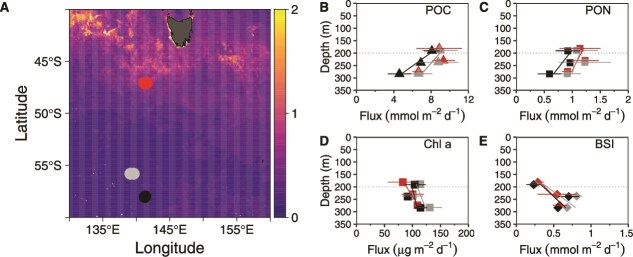
Southern Ocean study sites for surface-tethered free-drifting particle interceptor trap deployments. (A) austral summer mean (2020) satellite-derived chlorophyll-*a* data (NASA MODIS) overlaid with the locations of the three sampling sites. The subantarctic site (red symbol) was at the Southern Ocean time series (SOTS, 47^o^S, 141°E, [48]). Two sites were south of the polar front (gray symbol, PF1 (55^o^S,139°E) and PF2 (black symbol, 57^o^S,141°E, [47]). (B) the mean downward flux of POC (mmol C m^−2^ d^−1^), (C) mean PON flux (mmol m^−2^ d^−1^), (D) mean Chl *a* flux (μg m^−2^ d^−1^), (E) mean BSi (mmol m^−2^ d^−1^). Colored symbols represent the sampling sites in the subantarctic (red—SOTS and south of the polar front (gray—PF1; black—PF2)). Error bars denote the standard deviation of the mean for multiple deployments at each site. The horizontal dotted line represents the transition from the epipelagic (<200 m depth) and mesopelagic (>200 m depth) zones. Note, all sites were characterized by water columns with a < 2C decrease in temperature between the lower epipelagic and upper mesopelagic.

Samples were obtained during the SOLACE (Southern Ocean Large Area Carbon Export) voyage from 4 December 2020 to 15 January 2021. Surface-tethered free-drifting particle interceptor traps (pits) and c-respire dual particle interceptor/incubators (Fig. 1) were deployed repeatedly at three depths in the upper mesopelagic (S-Table 1) at one subantarctic (SOTS, Southern Ocean Time Series (47^o^S, 141°E), [46]) and two polar sites (Fig. 2A, (55^o^S,139°E and 57^o^S,141°E)). All sites were located in low advective regimes based on satellite maps of sea surface height anomaly [47]. Deployment depths differed slightly between sites to accommodate varying mixed layer depths (50 m SOTS, 65 m PF1, 75 m, PF2). Therefore, in the results the trap deployments are referred to as depth 1 (shallowest), 2 (intermediate) and 3 (deepest). Profiling robotic floats (https://biogeochemical-argo.org/) were deployed during SOLACE at SOTS and PF1, and provided ancillary data on particle dynamics (supplementary materials).

### Trap deployments

At each site, due to mooring design constraints, pit and c-respire traps were deployed ~20 m apart (Table S1), on the same array for *in situ* particle interception (Fig. S2) prior to incubation. Three types of pit tubes were prepared: (i) used a preservative (buffered-formalin brine) for analyses of POC, particulate organic nitrogen (PON), Biogenic Silica (BSi), and chlorophyll a [49]; (ii) had polyacrylamide gel in the base of the trap to observe particle characteristics using microscopy; (iii) had no preservative to obtain “fresh” sinking particles to subsequently determine ARQ shipboard. Upon recovery, pit tubes with preservatives or gels were processed (supplementary materials). A subsample of particles from pit tubes with no preservatives were incubated to measure ARQs. For the c-respire traps, a subsample of the postincubation particle assemblage was further incubated onboard to estimate the ARQ (Fig. S1 and S2). Subsamples of the seawater from c-respire were analyzed to estimate nitrification rates (Table S2) and DOC accumulation (supplementary materials).

### Sample analysis and apparent respiratory quotient determination

Pit tube C and subsamples of c-respire particles were transferred to a temperature-controlled lab (Table S3). Each tube was placed upright in darkness for 1 h for disturbed particles to re-settle (following transit to the lab). Then the overlying water was gently siphoned off. Particles were harvested and placed in 4 ml glass sample vials each containing a PreSens O_2_ and CO_2_ sensor and filled with 0.2 μm filtered sea water from the depth of deployment (Fig. S3). A subsequent lab-based experiment to map ARQs on a krill fecal pellet employed a modified optode system in conjunction with a flow chamber (supplementary materials).

### Statistics

A linear mixed-effects model was fitted to analyse ARQs, log-transformed to meet the assumptions of normality and homogeneity of variances. Fixed effects were tested by Type III ANOVA with Kenward–Roger degrees of freedom. The random-intercept was included to account for between-site variability but was not itself subjected to significance testing. Significant main effects for both depth (*F*(2, 345.4) = 5.3, *P* = .005) and trap type (pit or c-respire) (*F*(1, 349) = 113.3, *P* < 2.2 × 10^−16^), indicating that ARQ values were influenced independently by the depth of sampling and trap type. However, sampling sites (Fig. 2A) did not show a significant main effect (*F*(2, 4.6) = 0.57, *P* = .60), suggesting consistent ARQ values across sampling locations when accounting for depth and trap effects. Significant two-way interactions were detected between depth and trap type (*F*(2, 345.5) = 14.9, *P* = 6.11 × 10^−7^), and between depth and sampling location (*F*(4, 345.4) = 3.3, *P* = .09) demonstrating that the influence of trap type on ARQ, along with depth-specific patterns in ARQ, varied among site groups. In contrast, the interaction between trap type and site location was not significant (*F*(2, 346.4) = 1.4, *P* = .23).

Pairwise comparisons revealed significant differences between pit and c-respire (“r” vs. “s”) across epipelagic (<200 m) and upper mesopelagic (~220 m) for all sampling locations, with c-respire consistently yielding lower ARQs (all comparisons *P* < .01). At depths >250 m, significant differences between traps were observed only within SOTS (*P* < .0001), whereas PF1 and PF2 showed no significant difference. ARQ values from c-respire were ~30%–60% lower than for pits, with these differences most pronounced at shallower depths and decreasing progressively with depth. All analyses were performed using R software (v4.4.3).

## Results

### Upper Ocean characteristics

At the SOTS and PF2 sites, mixed layer depth was coincident with the vertical distribution of phytoplankton stocks (chlorophyll) [47, 50]. In contrast, at PF1 there was a 25 m thick deep chlorophyll maximum from 65 to 90 m depth [47]. In all cases, the shallowest trap depth was >90 m deeper (i.e. deployed at >180 m depth) than the maximum vertical extent of chlorophyll stocks.

### Particle flux attenuation and characteristics

Trends in flux attenuation at each site (Figs 2B–E) indicate how degraded the particle assemblage was at depths 2 and 3 (upper mesopelagic) relative to depth 1 (lower epipelagic). The vertical profiles were similar between sites, with ~25%–32% of the POC flux at depth 1 being attenuated by depth 3 (Fig. 2B). POC flux attenuation coefficients, calculated using Martin *et al.* [2], were lowest at SOTS (0.64 ± 0.31) and highest at PF2 (0.70 ± 0.36), indicating more mesopelagic flux attenuation at the polar sites (Table S4). The attenuation of PON flux was comparable to that of POC flux across all sites (Fig. 2B and C). In contrast, the BSi flux increased with depth at all sites, indicating a longer remineralization length-scale, a trend observed in other ocean regions and in global models [23] linked to BSi dissolution versus biologically mediated POC and PON flux attenuation [51]. Vertical chlorophyll a fluxes, potentially representative of more labile particles, were low (< 135 μg m^−2^ d^−1^) relative to POC fluxes (C:chla ratios of ~50–150, Supplementary Fig. S4). Because phytoplankton C:chla ratios are typically 10–30 [52] these mesopelagic chlorophyll fluxes indicate low proportions of pigment in the particle assemblage.

Particle typology revealed distinct differences between sites (Fig. S5). SOTS was dominated by small, dense aggregates containing a high proportion of zooplankton fecal pellets (likely to contain traces of chlorophyll-containing phytoplankton, Fig. S5B), which were also prevalent at PF1 but composed of larger and less dense fecal material, probably from salps [53]. In contrast, the PF2 assemblage revealed fecal pellets along with phytodetrital aggregates and intact diatom cells (Fig. S5; Fig. S13 in [47]). Trends in both particle flux attenuation and characteristics (Fig. 2) point to more degraded particles at depth, likely due to longer exposure to zooplankton- and microbially mediated transformations. Such degradation results in less intact pellets (i.e. loss of peritrophic sheath) evident from analysis of Southern Ocean mesopelagic pellets in polyacrylamide gel traps [54]. The vertical gradient in degradation in turn provides a spectrum of particles to investigate the temporal sequence of degradation using short- and long-term incubations of particles intercepted at each of three depths in the upper mesopelagic (Fig. 1).

Particle sinking speeds set the transit time from the upper epipelagic to traps deployed at SOTS, and therefore also contribute to their degradation timescale prior to their interception [26]. During SOLACE, transit times, based on based on a subset of 14 particle size-classes (that penetrated to at least 180 m depth) from a profiling robotic float, ranged from <1–7.6 days (sinking rates of 17 m d^−1^ to 136 m d^−1^ (supplementary materials), for depth 1, 0.5–4 days for depth 2 and 0.3–1.5 days for depth 3 (Table S6, and Ancillary data in S-Materials). Particle settling would therefore extend degradation timescales by <1–8 days, and sinking rate increases with depth (Fig. 2E). Petiteau *et al.* [49] used a combination of gel traps, modelling and UVP6 image analysis (all particles, suspended and sinking) to estimate sinking rates during SOLACE from 1–21 m d^−1^—corresponding to transit times of >70–5 days for all deployment depths. Several strands of evidence suggest that particles intercepted by pit and c-respire traps settled relatively rapidly, towards the lower bound of transit times. First, reported settling speeds of zooplankton fecal pellets (Fig. S5A) of >100 m d^−1^ are commonly reported [55]. Second, if particles were transiting the upper epipelagic to depth 1 in closer to 7.6 days (i.e. threefold longer than the 48 h long-term incubations), then much lower ARQs (see next section) would be anticipated.

### Apparent respiratory quotients from short- and long-term incubations

**Figure 3:**
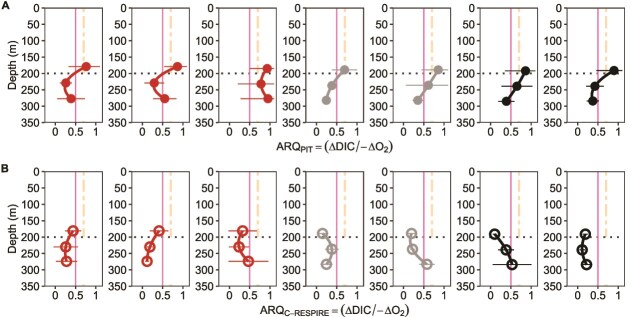
Apparent respiratory quotients obtained from shipboard incubations of “fresh” and degraded mesopelagic particles. (A) ARQ (ΔDIC/-ΔO_2_) of particles collected in particle interceptor traps (pit) at three depths (i.e. “fresh” particles obtained <72 h and > 0 h before trap recovery) then shipboard incubated for 12- h (B) ARQ of pre-incubated particles intercepted (i.e. <36 h > 0 h before in situ incubation) then incubated in situ using c-respire at depth for 36 h before being transferred (i.e. partially degraded particles) to shipboard incubators for a further 12 h incubation to determine ARQ. The horizontal dotted line represents the boundary of the upper- mesopelagic zone. The pink vertical line represents the theoretical lower limit of ARQ for full oxidation (0.5). The orange dashed vertical line represents an epipelagic ARQ of 0.7 calculated by Tanioka & Matsumoto [56]. Symbol colors represent the sampling location sites corresponding to Fig. 2A from subantarctic on the left to PF2 on the right. Each plot represents one deployment per site. Error bars are the standard deviation of the mean.

Short-term incubations of particle assemblages generally displayed a depth-dependent decrease in ARQ across all sites (Fig. 3A, Table S3). One exception was at SOTS, where there was no statistical difference between ARQs at depths 2 and 3 (Fig. 3A). ARQs for incubated particles from depth 1 were typically ~0.8 decreasing to 0.5 or less by depth 3 (Fig. 3A). Based on the statistical analysis, there was a significant difference in the ARQ of particles intercepted within the epipelagic zone (depth 1) compared to those from the upper mesopelagic (depth 2 and 3). Across these depth strata, the largest decrease in ARQ was observed at PF1 (47%), and PF2 (49%) and the smallest was at SOTS (37%). These results demonstrate that ARQ is not constant with depth (Fig. 3A) but decreases likely due to particle degradation as they settle (Fig. 2). Despite differences in the composition of particle assemblages (Fig. S4 and S5), no significant difference in ARQs was observed between sites for short-term incubations (Table S3).

In contrast to the short-term incubations, ARQs from the long-term incubations (Fig. 1) were significantly lower, with ARQs for the particles incubated from depth 1 of 0.5 or less (Fig. 3B). Unlike the decrease in ARQ with depth observed for the short-term incubations (Fig. 3A) there was no significant decrease in ARQ with depth in 6 of the seven profiles pointing to the approach of an endpoint of microbial degradation (Fig. 3B). The depth dependency of particle flux attenuation (Fig. 2) and wide-ranging ARQs from >0.7 (Fig. 3A) to ~0.2 (Fig. 3B) enables the temporal sequence of particle degradation to be further explored.

### Lab-based study to map apparent respiratory quotients

**Figure 4:**
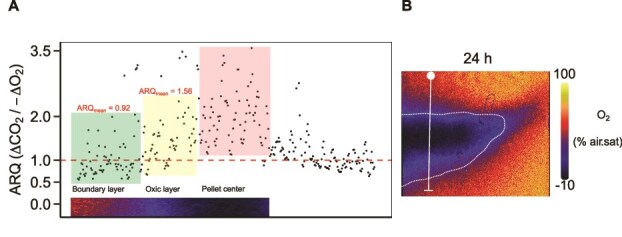
Exploring the range of ARQs using a 24 h laboratory incubation of an individual particle. (A) Map of ARQs across an intact krill faecal pellet (~0.55 mm width, denoted by white vertical line in panel (B). (B) Section across the pellet after 24 h reveals an anoxic Centre (blue pixels) and a hypoxic surrounding region (red/orange pixels). The ARQs were obtained using high resolution concurrent changes in CO_2_ and O_2_, rather than in DIC (c.f. Fig. 3) as it was not possible to convert CO_2_ to DIC for this experiment conducted in a flow cell.

To explore the range of potential ARQs in sinking particles we performed a subsequent study using an intact krill fecal pellet (i.e. enclosed in a peritrophic sheath). In the context of degradation, pellets are particularly interesting as they link particle transformations by flux-feeders and microbes, the dominant drivers of flux attenuation. ARQs (based on dCO_2_/dO_2_) exhibited a sevenfold range (0.5–3.5) over a 24 h incubation (Fig. 4) that was closely linked to their location within the pellet. The highest ARQs were at the anoxic center, whereas the lowest were at the periphery. These lab-based ARQ data are mainly higher than those observed at the field sites (Fig. 3). This trend is likely due to the oxic conditions in all *in situ* incubations (Table S5) along with the likelihood of porous (and hence oxic microenvironments, [57]) fecal pellets in the mesopelagic ([54]; Fig. S5 present study).

## Discussion

This investigation of trends in microbially mediated ARQs during particle degradation builds on the c-respire study previously published (Bressac *et al.* [9]. They explored depth-related patterns in the contribution of microbes to POC flux attenuation across six oceanic provinces and had to assume ARQs to convert their dissolved oxygen time-series to DIC. The findings of the present study also have implications for researchers who assumed constant RQs with depth to convert dissolved oxygen data from profiling robotic floats to carbon [58]. The present study reveals depth-dependent decreases in ARQs during short-term incubations, along with quotients <0.5 (i.e. below the theoretical minimum for complete oxidation) in long-term incubations. First, the trends in ARQs are compared with those from other studies. Second, these trends are explored using published findings on sequential changes in the stoichiometry of microbially altered POC substrates. Trends are further investigated by discussing the assumption of incomplete oxidation of substrates, and the underlying rationale for microbes to do so, in the latter stages of particle degradation. To conclude, a conceptual framework is used to contextualise these findings into what sets the endpoint to the sequence of degradation of POC flux in the mesopelagic.

### Comparison of apparent respiratory quotients with other studies

How do the ARQs for the particle assemblages at three Southern Ocean sites compare with those from other studies? The literature reports a wide range of ARQ and respiratory quotient (RQ) values (Table 1). A comparison across these studies is problematic as they use wide-ranging methods, metrics, particles from different depth strata, and in particular, suspended or an assemblage with both suspended and sinking particle types. In some cases they express RQs in terms of changes in dissolved oxygen relative to carbon ([59], 2020b, [60]) and/or use a mass balance approach which integrates over much longer timescales. Indeed, Anderson and Sarmiento [61] in their long-term (400–4000 m depth, years to centuries) basin-scale study state that the RQs they report may not be applicable on short timescales or length scales.

Only one study has investigated the depth-dependency of RQs [60] using an instantaneous chemical assay [59] and a mass balance approach on predominantly suspended particles from the epipelagic to 1000 m depth. Gerace *et al.* [60] noted a decrease in RQ (recast in Table 1 as (rΣ C: -O_2_)) from 1.00 to 0.71 which they ascribed to depth-dependent changes in planktonic community composition and preferential production/removal of biomolecules. The RQs at depth (to the base of the mesopelagic) are less than the ARQs from 48 h incubations (Fig. 3B). Due to the methodological differences between these two approaches a direct comparison of these findings is problematic, but both reveal depth-dependencies of ARQs and RQs in the mesopelagic.

**Table 1:** Summary of methods, metrics, and types of particles analysed in observations of ARQs and RQs. ^a^denotes estimating RQs using the relationship between oxygen demand and enhanced DIC and the elemental ratios of organic matter. ^b^denotes long-term quotients from the deep ocean that should not be compared with short-term quotients.

Method	Metric	Particle type/depth strata	Reference
Mass balance	RQ (RDIC/RO_2_) and other elements^a^ 0.69 for ocean basins	Dissolved nutrient ratios from >400 m depth	Anderson and Sarmiento [61]^b^
Incubation experiments	ARQ (RDIC/RO_2_) 0.3–1.8	Microbial epipelagic communities	Berggren *et al.* [62]
Incubation experiments	ARQ (RDIC/RO_2_) 0.3–3.5	Microbial epipelagic communities	Robinson [42]
Time-course experiments	RQ (RCO_2_/RO_2_) 0.2–9.6	Microbial lab cultures/epipelagic	Romero-Kutzner *et al.* [37]
Instantaneous chemical assay	Recast as RQ rΣC:-O_2_) i.e. the ratio of POC to Σ-O_2_ Typically around 0.8–1.0 for ocean basin transects	Suspended particles/epipelagic	Moreno *et al.* [59]
Pigments, biochemical theory, ocean colour	RQ (RCO_2_/RO_2_) 0.7 for detritus across much of the ocean	Suspended particles/epipelagic	Tanioka and Matsumoto [56]
Instantaneous chemical assay and a mass balance approach	Recast as RQ rΣC: -O_2_) i.e. the ratio of POC toΣ-O_2_ with depth 0.7–1.0	Suspended particles/epi and mesopelagic	Gerace *et al.* [60]^b^
*In situ* and shipboard incubations	ARQ (RDIC/RO_2_) 0.3–0.8	Sinking particles/mesopelagic	Present study

There are reports of differences in the dominant degradation processes for suspended versus sinking particles that may account for some of the contrasting depth-dependent trends observed by Gerace *et al.* [60] and the present study. For example, a metabolomic multisite South Atlantic study [16] revealed an enhancement of degradation products such as xanthine and glycine, from lipids and nucleic acids, in sinking relative to suspended (epipelagic) particles. Conversely, metabolites associated with phytoplankton processes comprised most of the pool in suspended relative to settling particles [16]. Resolving the depth-dependency of ARQs and RQs will assist studies which develop mesopelagic budgets of net community production by converting dissolved oxygen data from profiling robotic floats to carbon (e.g. [58]).

Others have explored the underlying reasons for wide-ranging ARQs (0.2–9.6, [37]) observed for marine bacterial cultures grown under acetate (lipid metabolism) and pyruvate (carbohydrate metabolism) replete and deplete conditions. They reported low RQ values under substrate-replete conditions, with a pronounced increase in RQ under starvation conditions due to changes in oxygen requirements decreasing before that of CO_2_. If these physiological trends are applicable to the present study (Fig. 3) they suggest that the resident microbes at the subpolar/polar sites were under substrate-replete conditions (see later section on what sets the limit for microbially mediated POC flux attenuation). In the present study, the seven-fold range of ARQs from detailed mapping of a krill fecal pellet was closely linked to the degree of anoxia and hypoxia (Fig. 4). These lab-based ARQs from the pellet center are several-fold higher than in the present study (Fig. 3) suggesting that the intercepted zooplankton fecal pellets (Supplementary Fig. 5) were not intact and therefore porous, consistent with other gel trap studies [54], resulting in lower ARQs (Fig. 3 c.f. boundary layer ARQs in Fig. 4). Thus, in addition to substrate quality/degree of oxidation, and metabolic pathway [36], ARQs are influenced by oxygen concentration (Fig. 4) and physiological status [37]. Changes in microbial community structure, physiology and biochemical substrates with depth [63] and their degree of oxidation [24] point to the potential for wide-ranging ARQs, on a timescale of days, for sinking particles throughout the mesopelagic.

### Biochemical interpretation of ARQ trends

Careful interpretation of ARQs provides insights into the nature of the organic matter being oxidised, and the degree of oxidation. Prior to employing this interpretation, the influence of other metabolisms (nitrification, calcification and anaerobic pathways), must be corrected for [36]. Anaerobic pathways are highly unlikely (Table S5, Fig. S5A), the minor effects of nitrification have been used to recalculate ARQs (Table S2), and based on phytoplankton assemblages at each site, calcification was not an issue (supplementary methods).

There is a well-established linkage between altered substrate quality and ARQs that reflects the stoichiometry of (complete) oxidation of the substrate. For example, glucose has an ARQ of 1 (6 CO_2_:6 O_2_, whereas phytoplankton based on their chemical composition, should have an ARQ of ~0.89, and lipids such as fatty acids have an ARQ of 0.7 [42]. The observed decreases in ARQ from short-term incubations with depth are consistent with microbially mediated changes in particle biochemistry with vertical flux attenuation (Fig. 2 c.f. Fig. S5), indicative of altered substrate quality of particles. The theoretical minimum for complete substrate oxidation is 0.5 for methane [36], which is utilised by specialist methanotrophs [64]. In the long-term incubations (Fig. 3B  c.f.  A) there was a decrease in ARQs, indicative of a threshold in the transformation of particles when they are subjected to increasing degradation. This threshold may be linked to observed values of <0.5 pointing to the role of partial oxidation of substrates by microorganisms (i.e. a decrease in oxygen but no corresponding increase in DIC). Underlying rationale for partial oxidation is explored next.

### The role of the degree of oxidation in setting ARQs

In the present study, trends in ARQs are indicative of a sequence of particle degradation with depth, and with time (Fig. 1). However, their lower bound, in particular for the long-term incubations (Fig. 3B), is <0.5. Others have reported ARQs of <0.5 [37, 42] for marine microorganisms but did not provide explanations. Although the partial oxidation of substrates can also control the magnitude of ARQs [36], the wider ramifications of this pathway require several questions to be addressed for it to offer a compelling mechanism for the observed low ARQs. What are its physiological and ecological ramifications and does the observed lower limit for ARQs (~0.2, Fig. 3B) control the magnitude of the sinking POC flux i.e. degraded by marine microorganisms?

Investigations of a free-living SAR202 community have hypothesized the potential role of partial oxidation in particle degradation [65, 66], and there is emerging evidence for partial oxidation by microorganisms from DOC incubation time-series [67]. These findings lead to additional lines of enquiry in relation to the present study. What is the fate of microbially degraded POC and why would microorganisms only partially oxidize substrates? Studies of ocean biogeochemistry interchangeably use terms such as particle remineralization, breakdown, transformation or degradation [9] but seldom detail the process of degradation [39] is an exception). It is well-established that virtually all POC must first be broken down, often via solubilization by exoenzymes, to low molecular weight compounds [68–70]. This is a pre requisite to uptake via bacterial ABC (ATP-Binding Cassette) transporters into the cell. So, the microbially mediated fate of POC is selective solubilization to DOC followed by transport into the attached bacterium with some carbon converted into biomass. This sequence is presumably repeated – leading to a substrate continuum of POC as it is continually being microbially transformed—during the temporal sequence of particle degradation across different substrates and hence enzymes.

Explanations for “why would microorganisms only partially oxidize substrates?” add both a physiological [71] and ecological [71, 72] rationale for this oxidative pathway. Gralka *et al.* [71] reviewed the concepts underlying microbial community structure and the associated trophodynamics. They report that “*Incomplete metabolism of the primary substrate and the excretion of metabolites has been observed widely in microbes, including proteobacteria, firmicutes, actinobacteria, and yeasts, from a variety of environments, making it a likely widespread feature of bacterial (and fungal) metabolism*.” They discuss why microbes (which they term primary degraders, the first “responder” to the availability of complex substrates, such as on sinking particles) would not fully oxidise the carbon source even though they may have that capability.

Gralka *et al.* [71] propose that imbalances in microbial metabolism, centred around anabolism and catabolism, leads to a leakage of metabolic intermediates. They provide supporting evidence of this leakage mechanism from the so-called overflow metabolism exhibited by *Escherichia coli*. This physiological mechanism is then linked to microbial ecology, because the leaked biochemical intermediates can be utilised by other microorganisms that Gralka *et al.* [71] term secondary consumers as they have a niche to exploit linked to the consumption of the intermediates. The authors go on to link this leakage and the resulting availability of diverse substrates, for the consortia of primary degraders and secondary colonisers (some of whom may have sharing versus selfish modes of substrate utilization, Reintjes *et al.* [73, 74]), as a means to increase microbial diversity and succession during the degradation process. These patterns have been reported in modelled microbial community studies [75, 76]. Furthermore, Zakem *et al.* [72] have built on this concept of Gralka *et al.* [71] by linking multiple DOC pools (a continuum of different reactivities) and their interplay with diverse microbial populations (i.e. a spectrum of generalists to specialists, Fig. 1 (Zakem *et al.* [72]).

The physiological (overflow metabolism) and ecological (community structure and stability) advantages of partial oxidation point to the benefits of this metabolic strategy. However, it remains unclear how partial oxidation fits into the temporal sequence of POC degradation (later section on the conceptual model) evident from the decrease in ARQs from ~0.7 to <0.5–0.2 (Fig. 3B). Specifically, is the sequence of particle degradation—evident from trends in ARQ—driven initially by complete oxidation of POC substrates followed by partial oxidation of DOC along a substrate continuum? Or, given the physiological and ecological advantages of partial oxidation, are these two distinctive transformations interspersed over the timescales of degradation? In terms of a sequence of degradation, partial oxidation of DOC likely represents the limit of microbially mediated POC transformations [67].

### What sets the limit on particulate organic carbon degradation by microbes?

Based on microbially mediated respiration using c-respire, Bressac *et al.* [9] report that microbes drive 7%–29% of POC flux attenuation in the upper mesopelagic across six different oceanic provinces. To understand the role of microbes in particle flux attenuation requires a conceptual framing of what sets their limit in POC degradation. Bressac *et al.* propose that temperature mainly controls particle flux attenuation at sites with vertical temperature gradients >2°C (mainly at low latitudes) in the upper mesopelagic. In contrast, at high latitudes, characterized by water columns with <2°C mesopelagic gradients, other factors, such as to substrate quality, may exert more complex controls on particle degradation.

The main trends of the present (high latitude) study concur with Bressac *et al.* [9] as they reveal the importance of changes in substrate quality during particle degradation, and point to additional complexity in controls—partial oxidation of DOC. ARQs of 0.2 (Fig. 3B) may represent the lower bound of microbially mediated POC degradation, such that DOC solubilized from particles and/or microbially transformed from DOC precursors is in a form that cannot be further degraded by the resident particle-attached microbial assemblage in the upper mesopelagic. Indirect evidence of such an end-point in degradation would be the accumulation of DOC (Fig. 2 in the model of [72]).

There is evidence of net DOC accumulation in c-respire after 36 h incubations (Fig. 6B) that correspond to low ARQs in parallel studies (Fig. 3B) and to a plateau in dissolved oxygen consumption being approached (Fig. 6A). Other studies have reported that excess DOC was present within a wide range of settling particles at a coastal Californian site (Alldredge [17]. This study reported that DOC comprised up to 30% of POC + DOC in each aggregate and was likely driven by solubilisation. Alldredge [17] reported 100-fold DOC gradients (relative to the surrounding seawater) that were maintained by physical processes (the fractal maze of particles and low diffusion rates). However, the research did not explore whether DOC accumulation was due to the inability of the resident microorganisms to further degrade it. Such accumulated DOC within particles has two fates—either consumption by specialist microorganisms deeper in the water column [77, 78] or alternatively forming part of the refractory DOC pool via the microbial carbon pump [79].

### Towards a conceptual model of sequential particle degradation

**Figure 5:**
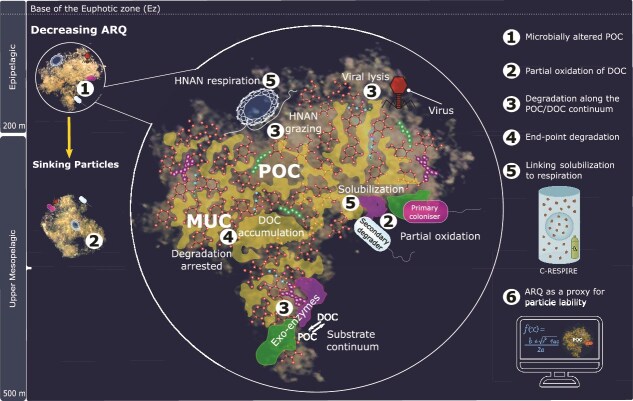
A conceptual model of the sequence of marine particle degradation from the lower epipelagic (i.e. depth 1) to the upper mesopelagic (i.e. depth 2 and 3) based on temporal and depth-related changes in the ARQ in the present study, and insights from other publications. (1) denotes depth-dependent changes in POC substrate reactivity linked to ARQ, due to microbial needs and substrate-driven microbial succession. (2) denotes physiological flexibility and ecological stability conferred by partial oxidation of DOC substrates. (3) denotes POC to DOC (exoenzymes) to bacterial carbon to DOC (lysis, bacterivory by heterotrophic Nanoflagellates (HNAN)) repeated cycling during particle attenuation, with implications for the continuum of POC and DOC reactivity. (4) denotes the factors that set the limit on POC (such as the molecularly uncharacterized component (MUC)) and DOC reactivity and hence degradation (as evidenced by DOC accumulation, Fig. 6B). (5) denotes the linking the energetics of microbial solubilisation of POC (exoenzymes and transporters) relative to respiration (key metric in c-respire) and hence to ARQs. (6) denotes the link to models of microbially mediated POC flux attenuation using ARQs.

The three prior subsections explored the interpretation of the trends in ARQ as: sequential changes in the stoichiometry of the microbially altered POC substrates, then the potential role of partial oxidation of DOC in degradation, and the drivers of the endpoint of this sequence of degradation. Here, a conceptual model is developed firstly as a framework to unravel the interplay between POC and DOC, during the temporal sequence of POC degradation, to explore how partial oxidation influences ARQ. Secondly, it is used to examine the following question: as degradation is largely driven by enzymatic solubilization of particles [68], can it be linked to respiration and hence to the c-respire method and the ARQ approach? If this link can be made, it may enable ARQ to be introduced as a metric into models to reflect the degree of lability in particles, as used by Nguyen *et al.* [26]. The model is therefore split into two components – a four-part mechanistic framework around degradation, followed by a two-part strategy to link these mechanisms to the interpretation of trends in microbially mediated POC flux attenuation through more fieldwork and modelling (Fig. 5).

The alignment of vertical patterns in particle flux attenuation and ARQ (Figs. 2 and 3A) along with evidence of particle transformations (Fig. S5) are consistent with the central role of POC degradation in setting the magnitude of ARQs. Conceptually, this explanation of microbially altered POC is indicative of changes in POC substrate quality driven by microbial metabolic needs [25] and/or substrate-driven microbial succession [80]. The marked decreases in the ARQs in the long incubations to values <0.5, with little change with depth (Fig. 3B) suggests a shift from particulate to dissolved transformations in setting ARQs (Fig. 5). Such changes in ARQs with depth may also reflect the use by microorganisms of partial oxidation of substrates for DOC [67]. The lab-based findings of Romero-Kutzner *et al.* [37] that lower ARQs are associated with substrate-replete microorganisms also needs to be considered in relation to complete versus partial oxidation, but this is presently beyond the scope of this conceptual model.

A major unknown that exists when considering controls on ARQs on POC flux with depth is whether these two controls are sequential (i.e. complete oxidation of POC substrates followed by partial oxidation of DOC) or interspersed? If the benefits of partial oxidation are major [71], then it could be the norm for both POC and DOC microbially mediated degradation. The interpretation of the findings of Fig. 3 is currently limited to stating that partial oxidation of DOC may follow after ARQs decrease below the threshold for complete oxidation of 0.5. However, the relationship between POC and DOC during particle degradation is more complex, with implications for the influence of degree of oxidation on ARQs.

**Figure 6:**
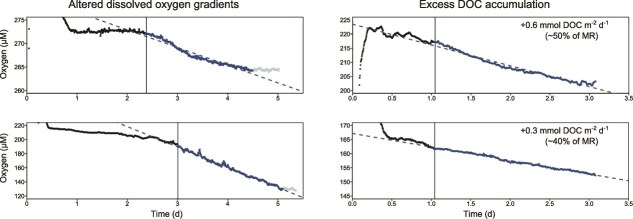
Dissolved oxygen (DO) time-series measured within the incubation chamber of the c-respire. The black vertical lines represent the switch between the particle collection (black symbols) and incubation phases (dark and light blue symbols). The dashed lines represent the best fit of the linear model obtained for the incubation phase (dark blue symbols). (A) Examples of DO time-series where a plateau is reached before the end of the incubation (light blue symbols). (B) Examples of incubations where DOC accumulation was observed. Rates of DOC accumulation over the incubation period (i.e. DOC concentration at the end of the incubation minus DOC concentration at time zero) are provided. MR denotes microbial respiration measured in the c-respire, and DOC accumulation is expressed as a proportion of MR.

The concept of a repeated sequence of POC degradation followed by DOC transformation is explored in Fig. 5. The pivotal role of solubilization in particle degradation points to a sequence of POC (particle) to DOC (via exoenzymes) to POC (intracellular DOC transport leading to bacterial growth) to DOC (viral lysis/bacterivory) to POC (bacterivore carbon and/or the microbially transformed particle) driving a diverse cascade of processes that may continually alter the reactivity or quality of the substrate (POC and/or DOC) providing new opportunities for microbes, as modelled by Zakem *et al.* [72]. There is indirect evidence of such a POC/DOC sequence from exoenzymes/ABC transporters (see earlier), video footage of particle colonization by grazers (supplementary materials in [23]) and experimental evidence, using ^14^C labelled DOC [43], of the complex interplay between DOC and POC in fecal pellets being driven by both physical (diffusion) and biological (microbial degradation) processes. The endpoint to such a reactivity or substrate continuum to describe degradation is determined by what factors set the limit to POC and DOC reactivity (Fig. 5). POC reactivity may be halted by the refractory nature of MUC (molecularly uncharacterized component, [81]) reported to be putative cell-wall like material of plankton and microbes [82]. For DOC, its accumulation ([17]; Fig. 6A) provides indirect evidence of its functional recalcitrance (sensu [72]).

The next challenge is to link this four-part degradation sequence to mesopelagic biogeochemical experimental (e.g. [9]) and community ecological modelling (e.g. [26]) approaches. The currency used in c-respire e.g. is respiration, and so the conceptual model explores how to link respiration to the energetics of solubilization (exoenzymes) and to also consider ways in which respiration and solubilization could be decoupled (such as by bacterivore respiration on sinking particles, Fig. 5). Solubilization requires energy to synthesize exoenzymes and to produce transporters to subsequently move dissolved substrate(s) into a bacterium [83]. These energy requirements provide a potential link to respiration, but there are confounding issues including the need to track solubilization energetics across the many exo-enzymes involved in degradation (Fig. 5). Perhaps the most comprehensive investigation of the marine bacterial respiration and solubilization relationship is the nearshore study of Shi *et al.* [84] who reported a correlation at the cellular level. In contrast, most mesopelagic studies cannot obtain a representative sample of different exo-enzymes [85] to fully explore the nature of the solubilization versus respiration relationship.

In the case of community ecological models, conceptually, the observed decrease in ARQ—that tracks the temporal sequence of microbially mediated degradation (Fig. 3) —is likely driven by solubilization of targeted substrates by the community of primary microbial degraders, secondary and other levels of bacterial consumers. The type of approach outlined in Fig. 5 can be linked to microbial community ecology in models such as Nguyen *et al.* [26] or Zakem *et al.* [72]. The final part of the conceptual model relates to how to bring ARQ as a metric into models to reflect the degree of lability in particles (as used by [26]). To do so, more knowledge on what permutations of full versus partial oxidation of substrates (POC to DOC to microbial POC, Fig. 5) set the ARQ and how they are driven by cellular physiological and/or particle community assemblage ecology [71].

There are already promising advances that provide linkages between some of the components (Fig. 5). For example, a study on marine microbes and the different components of DOC makes an important connection between ARQ, NOSC (Nominal Oxidation State of Carbon) and the thermodynamic analysis of degradation [67]. A study of the particle assemblage in the mesopelagic off Hawaii made cumulative estimates using NOSC to assess how oxidised and energy-deficient the particles are with depth [24]. The use of these metrics moves the marine field closer mechanistically to that of soil microbiology [30], however issues remain in terms of better understanding the composition of POC [81]. Such an understanding would help in the construction of a biochemical sequence of degradation that can be and linked to thermodynamic metrics, NOSC and potentially ARQ. The latter is a potentially powerful tool to employ in the oceans’ “twilight zone especially if it can be coupled with a series of metrics that would subsequently allow” them to feed into new microbial models which link specific microbial redox reactions with that of microbial functional types [86].

## Supplementary Material

Supplementary_Figures

Supplementary_materials_methods

Supplementary_Materials_wraf255

## Data Availability

The data underlying this article is available in the IMAS Metadata Catalogue (10.25959/2jd1-hv68).
