# Supplementary material for: Respiratory quotients of particle-associated microbes track carbon flux attenuation in the mesopelagic Southern Ocean
Source: ISME J. 2025 Nov 20;19(1):wraf255. doi: 10.1093/ismejo/wraf255 (PMC12694406; doi:10.1093/ismejo/wraf255)
Supplement: Supplementary_Figures [file supplementary_figures.pdf]

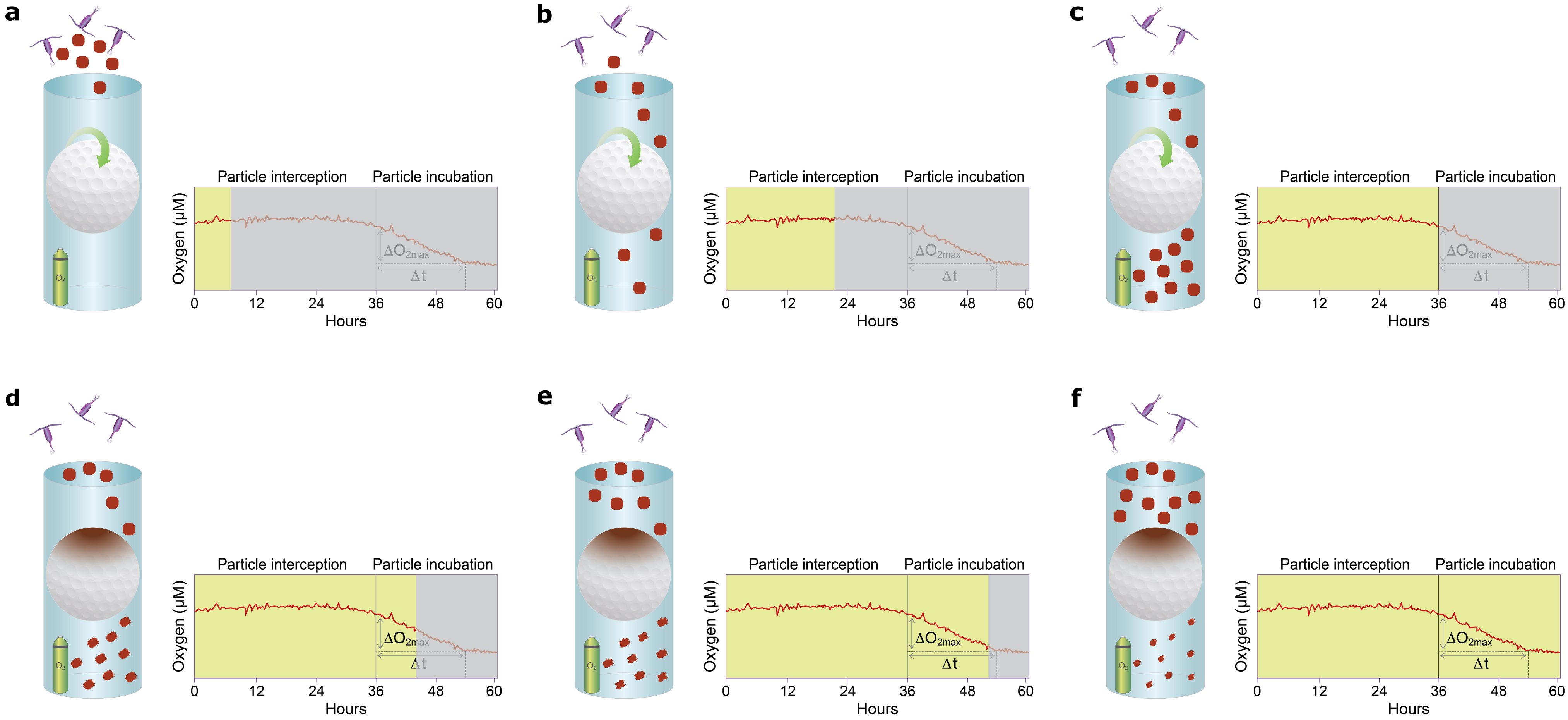
a
Particle interception
Particle incubation
Oxygen (μM)
ΔO2max
Δt
0 12 24 36 48 60
Hours
b
Particle interception
Particle incubation
Oxygen (μM)
ΔO2max
Δt
0 12 24 36 48 60
Hours
c
Particle interception
Particle incubation
Oxygen (μM)
ΔO2max
Δt
0 12 24 36 48 60
Hours
d
Particle interception
Particle incubation
Oxygen (μM)
ΔO2max
Δt
0 12 24 36 48 60
Hours
e
Particle interception
Particle incubation
Oxygen (μM)
ΔO2max
Δt
0 12 24 36 48 60
Hours
f
Particle interception
Particle incubation
Oxygen (μM)
ΔO2max
Δt
0 12 24 36 48 60
Hours

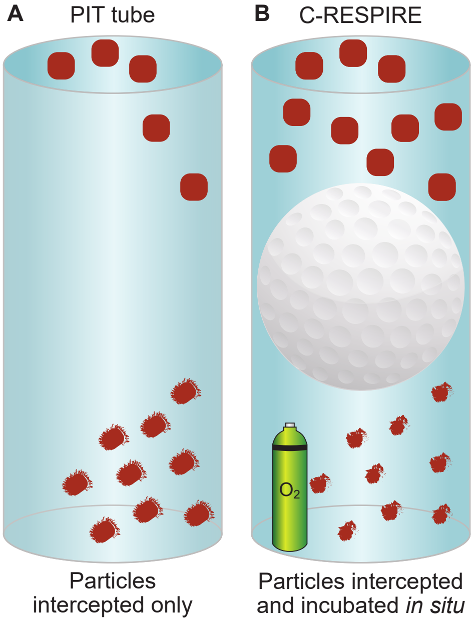
A
PIT tube
B
C-RESPIRE
O_2
Particles intercepted only
Particles intercepted and incubated *in situ*

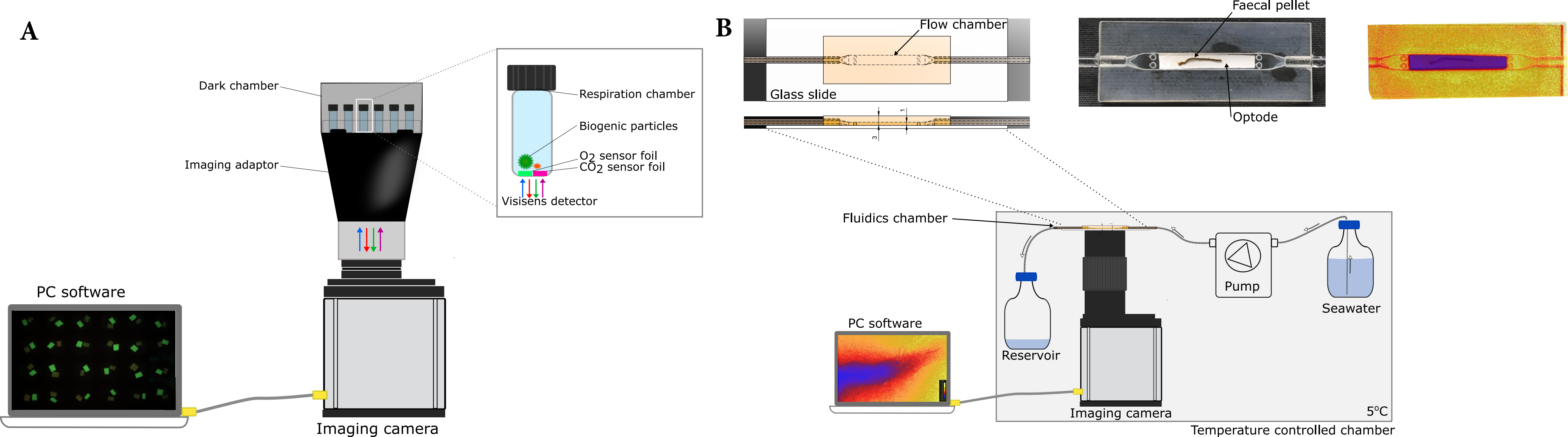
A
Dark chamber
Imaging adaptor
Respiration chamber
Biogenic particles
O2 sensor foil
CO2 sensor foil
Visisens detector
PC software
Imaging camera
B
Flow chamber
Glass slide
Faecal pellet
Optode
Fluidics chamber
Pump
Seawater
Reservoir
PC software
Imaging camera
5°C
Temperature controlled chamber

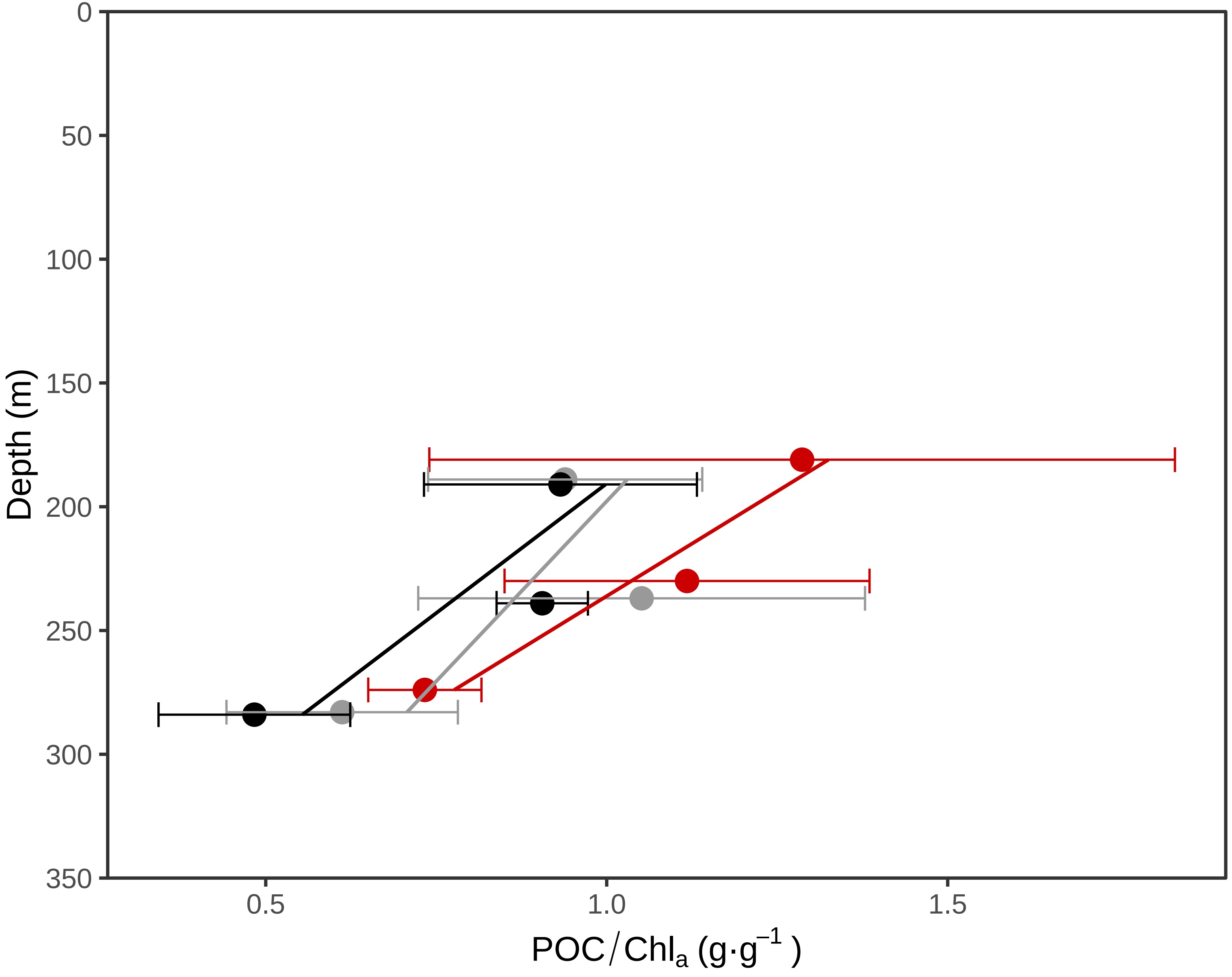

0
50
100
150
200
250
300
350
Depth (m)
0.5
1.0
1.5
POC/Chl$_a$ (g·g^{-1})

A

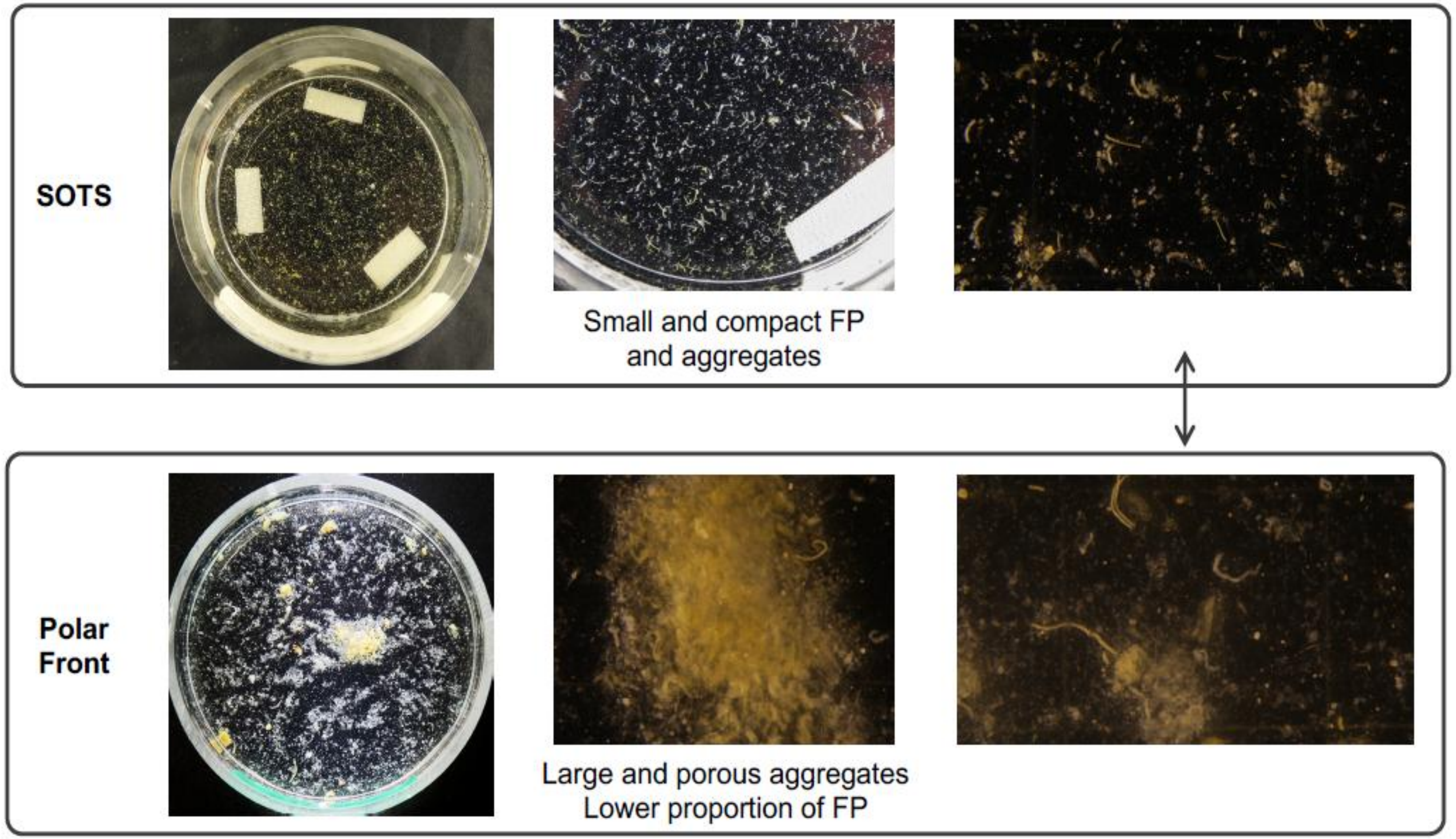
SOTS
Small and compact FP
and aggregates
Polar
Front
Large and porous aggregates
Lower proportion of FP

B

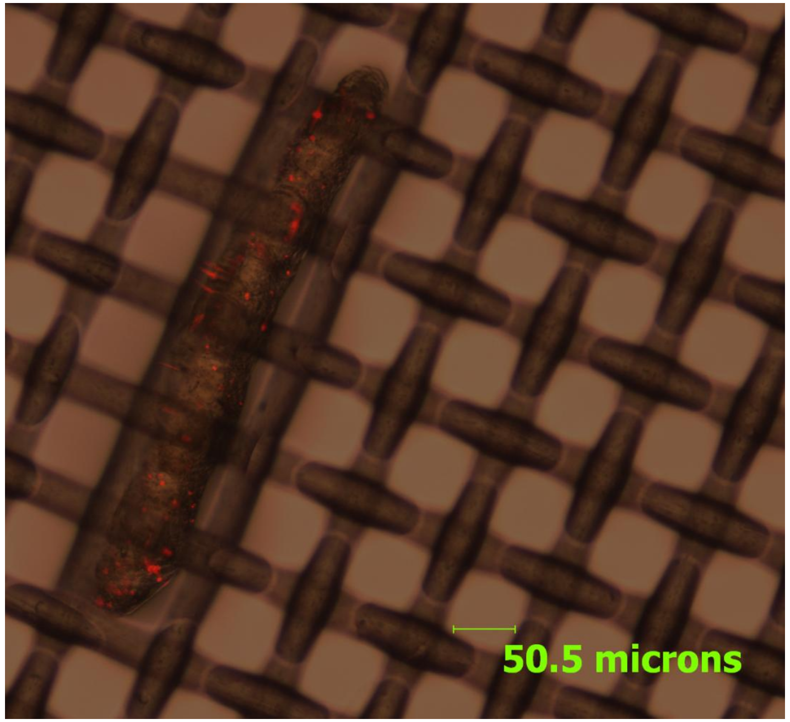
50.5 microns
