# Supplementary material for: Respiratory quotients of particle-associated microbes track carbon flux attenuation in the mesopelagic Southern Ocean
Source: ISME J. 2025 Nov 20;19(1):wraf255. doi: 10.1093/ismejo/wraf255 (PMC12694406; doi:10.1093/ismejo/wraf255)
Supplement: Supplementary_materials_methods [file supplementary_materials_methods.pdf]

Supplementary Methods

Respiratory quotients of particle-associated microbes track carbon flux attenuation in the mesopelagic Southern Ocean

Detailed methods

Gel trap methodology

One PIT tube per deployment depth contained a polyacrylamide gel jar (~3 cm thick) that was used to collect and image intact sinking particles. The method to analyze the gel samples is fully described in Petiteau et al. (in revision). Briefly, particles within the gels were photographed using a Zeiss Stemi 2000-CS stereomicroscope coupled to a Leica DFC-280 1.5MP digital camera with a magnification of ×6.5. A laser-etched glass grid of 36 cells was used for size calibration. The images were analyzed for particle counting, size, and shape evaluation using ImageJ, public domain Java image processing program developed by the U.S. National Institute of Health (Schindelin et al., 2012). The Equivalent Spherical Diameter (ESD) was calculated based on the projected area. Based on their ESD, particles were binned into logarithmically spaced size classes. Size class bins with less than 5 particles counted were considered under sampled and removed from the dataset. Numerical particle fluxes were then calculated as the number of particles in each size class divided by the deployment duration and trap collection area (Ebersbach & Trull, 2008).

Ebersbach, F. and T.W. Trull (2008) Sinking particle properties from polyacrylamide gels during the KErguelen Ocean and Plateau compared Study (KEOPS): Zooplankton control of carbon export in an area of persistent natural iron inputs in the Southern Ocean. Limnol. Oceanogr., 53(1), 2008, 212–224.

Schindelin, J., Arganda-Carreras, I., Frise, E., Kaynig, V., Longair, M., Pietzsch, T., Preibisch, S., Rueden, C., Saalfeld, S., Schmid, B., Tinevez, J.-Y., White, D. J., Hartenstein, V., Eliceiri, K., Tomancak, P., & Cardona, A. (2012). Fiji: An open-source platform for biological-image analysis. *Nature Methods*, *9*(7), 676–682. https://doi.org/10.1038/nmeth.2019

Correcting for nitrification rates and calcification

The C-RESPIRE traps also provide estimates of nitrification rates. NO_2 (along with NH_4 and NO_3) concentrations were measured prior to the deployment (i.e., ambient concentrations) and at the end of the incubation (S-Table 1). In the incubation chamber, both denitrification and nitrification likely occurred simultaneously, so only residual nitrification rates can be estimated using the delta NO_2 (i.e., $NO_{2\ \text{post-incubation}} – NO_{2\ \text{pre-incubation}}$). At our study sites and depths, these rates ranged between 3-30 nmol N L^{-1} d^{-1}. Nitrification rates were used to amend the ARQ estimates so that the only metabolic pathways considered were dDIC/DO_2 (calcifiers were a minor presence in the phytoplankton assemblage over the annual cycle at SOTS (Erikson et al., 2017) and diatoms dominated at the polar sites (Boyd et al., 2024)). All ARQs were corrected for nitrification (<15% at all sites and depths) using datasets collected concurrently in the upper mesopelagic from C-RESPIRE (see Supplementary Table S3) in conjunction with a delta NO_2/delta O_2 stoichiometry of 4.23 mg O_2/mg-N oxidized for complete nitrification used for the ARQ correction. This value was derived from a pulse flow respirometer used to measure the oxygen uptake for complete nitrification with activates sludge samples for an aquatic system (Liu and Wang, 2012).

Boyd, P.W. et al. (2024) Controls on Polar Southern Ocean Deep Chlorophyll Maxima: Viewpoints From Multiple Observational Platforms. Global Biogeochemical Cycles. https://doi.org/10.1029/2023GB008033

Eriksen R, Trull TW, Davies D et al. Seasonal succession of phytoplankton community structure from autonomous sampling at the Australian Southern Ocean time series (SOTS) observatory. Mar Ecol Prog Ser 2018;589:13–31. https://doi.org/10.3354/meps12420

Liu, G. and J. Wang (2012) Probing the stoichiometry of the nitrification process using the respirometric approach. Water Research, 46, 5954-5962. https://doi.org/10.1016/j.watres.2012.08.006

DOC accumulation during C-RESPIRE incubations

Net accumulation rate of dissolved organic carbon (DOC) within the C-RESPIRE during the 36-h in situ incubation were obtained by comparing pre- (i.e., ambient) and post-incubation concentrations (i.e., in the C-RESPIRE incubation chamber). These rates, not measured during SOLACE, were quantified during deployments in the oligotrophic South Pacific (Bressac et al. 2024), and HNLC North Pacific (A. Santoro, pers. comm.). At both sites, DOC accumulation rates ranged between 0-0.6 mmol m^{-2} d^{-1} and were highest in the North Pacific.

Water samples for DOC analysis were immediately filtered under a low vacuum (<50 mmHg) through pre-combusted (450°C, 6 h) glass fiber filters (GF/F, ~0.7 mm, 25 mm diameter, Whatman) using all-glassware systems. Samples were stored frozen until analysis. DOC was determined by high-temperature catalytic oxidation using a Shimadzu TOC 5000 Total Carbon Analyzer (Kyoto, Japan).

Sensor calibration, calculation of ARQs and conversion of CO_2 to DIC.

Sensor calibration was conducted following the manufacturer's instructions. The O_2 sensors were standardized using a two-point calibration (0, 100% air sat), and the CO_2 sensors were calibrated using a 6-point calibration with specific gas mixes (0-5%) (BOC Linde gas). We could only measure CO_2 and therefore cannot consider any subsequent interplay with other components of the carbonate chemistry.

Sample images were processed using PreSens IDL evaluation software. Images obtained over 12 h were used to determine particle ARQs. Oxygen consumption (mol O_2) and CO_2 production (mol CO_2) during incubation was obtained as follows:

$$dO_2 = ((O_{2\,\text{Pre-incub.}} - O_{2\,\text{Post-incub.}}) - (O_{2\,\text{CONTROL Pre-incub.}} - O_{2\,\text{CONTROL Post-incub.}}))$$

$$dCO_2 = ((CO_{2\,\text{Pre-incub.}} - CO_{2\,\text{Post-incub.}}) - (CO_{2\,\text{CONTROL Pre-incub.}} - CO_{2\,\text{CONTROL Post-incub.}}))$$

The values obtained were used to calculate the ARQ of each sample vial, where,

$$RQ = dCO_2/-dO_2$$

It is important to acknowledge that conducting the experiment shipboard may introduce factors that influence the rate of microbial respiration (Amano et al., 2023).

Amano, C. et al., (2023) A device for assessing microbial activity under ambient hydrostatic pressure: The in situ microbial incubator (ISMI). Limnol. Oceanogr. Methods 21, 69–81

Conversion of CO_2 to DIC

DIC concentrations were estimated using calculated partial pressure of CO_2 (pCO_2) values. The change in DIC (ΔDIC, in µmol/kg) was determined using the relationship:

$\Delta DIC = 0.18 \times \Delta pCO_2$

The variability in DIC and pCO_2 was assessed across different sites, with particular attention to the 47°S, 56°S, and 58°S locations. The spread in the observed data was influenced by differences in in-situ temperatures and salinities. The incubation temperatures for the samples ranged between 4°-6°C, while in-situ temperatures were lower, ~1°-2°C. Salinity variations were also considered, with the 56°S and 58°S sites exhibiting the lowest ranges. Revelle factors, which describe the fractional change in pCO_2 relative to the fractional change in DIC, were calculated for different oceanographic zones. For the sites, Revelle factors were determined across a range of likely salinities (33.9-34.1) and temperatures (4°-6°C). Total Alkalinity (TA) was normalized to a value of 2335.1 ± 1.1 µmol/kg (n=7) at S=35, based on data from the 2018 SR3 hydrographic section (55.5°S–58.5°S, 200–300 m depth). DIC values used in the calculations ranged between 2260 and 2240 µmol/kg, consistent with measurements from the same hydrographic section.

Ancillary data – oxygen time-series and particle sinking speeds

To further interpret the ARQ datasets requires data on both the magnitude of oxygen consumption during the C-RESPIRE incubations and on the sinking rates of particles (Figure 1). S-Table 5 reveals that over the 7 deployments, oxygen consumption was typically <6.5% and in 2 cases was ~18% (SOTS deployment 2 – depths 2 and 3). Oxygen concentrations at the end of all 21 incubations were >192 µmol L^{-1} (the threshold for hypoxia is 62.5 µmol L^{-1}, Hofman et al., 2011). For the deckboard incubations the manipulations of particles and addition of filtered seawater from the corresponding depths of where the particles were sampled would have resulted in oxic conditions in all subsequent 12 h duration lab-based incubations.

Hofmann, A.F., E.T. Peltzer, P.M. Walz, P.G. Brewer (2011) Hypoxia by degrees: Establishing definitions for a changing ocean. Deep Sea Research I, 58, 1212-1226 https://doi.org/10.1016/j.dsr.2011.09.004

Sinking rates for the particle assemblage were derived using observations from a profiling robotic BGC-ARGO float, that had an Underwater Vision Profiler (UVP) particle imager (Picheral et al., 2022) deployed on a 4 year mission near the SOTS site during the SOLACE voyage (Lacour et al., submitted). S-Table 6 presents sinking rates and particle penetration depths for 14 particle size classes (0.11-2.31 mm Equivalent Spherical Diameter (ESD). Only particles >0.58 mm penetrated to 180 m (see S-Table 1). Thus, it is possible to constrain the transit time for particles at SOTS from the base of the 50 m mixed layer to depth 1 at ~180 m. Sinking rates range from 17 m d^{-1} to 136 m d^{-1}, corresponding to transits of 7.6 to <1 day). From depth 1 to depth 2 (~70 m) the sinking rates are 17-136 m d^{-1} (transits of 4-0.5 days) and to depth 3 (~50 m) sinking rates are 33-136 m d^{-1} (transits of 1.5-0.3 days).

These rates from SOTS can be cautiously applied to the other sites to constrain the transit times to depths 1-3 from the base of vertical chlorophyll distribution. The profiling float at SOTS (Lacour et al. submitted) along with another deployed at PF1 (see Figure 10, Boyd et al., 2024) during SOLACE (no UVP) also provides evidence of the constancy of the particle flux (based on backscatter) over the sampling period during the SOLACE voyage.

Lacour, L. et al. (submitted) Particle size sets the magnitude of the downward and return pathways of the Subantarctic biological pump. PNAS.

Picheral, M. et al. (2022) The Underwater Vision Profiler 6: an imaging sensor of particle size spectra and plankton, for autonomous and cabled platforms. L&O Methods, https://doi.org/10.1002/lom3.10475

Krill aquarium and pellet collection

In a shore-based lab study, faecal pellets from Antarctic krill (*Euphausia superba*) were sourced from aquaria at the Australian Antarctic Division, Tasmania. The krill were housed in large, temperature-controlled aquaria set at 0.5°C. These individuals were initially collected during austral summer 2017–2018 from polar Southern Ocean (60°S) waters, and had been kept in aquaria for three years prior to conducting experiments (2020–21).

Following collection, faecal pellets were placed within a transparent, 3D-printed flow chamber (3 mm x 20 mm) equipped with oxygen and pH optodes (Presens, Germany) within 90 minutes (Supplementary Figure S3B). Each pellet was positioned in a separate flow chamber and allowed to settle onto the optode surface over the course of one hour. The chambers were then sealed with silicone adhesive, and a glass microscope slide was affixed to the surface. A peristaltic pump was used to maintain a steady flow of chilled seawater, from the krill aquarium, through each chamber without recirculation. Seawater temperature was regulated at 5°C, slightly above the krill aquarium to ensure optimal performance of the optodes and accurate measurement of oxygen levels.

Oxygen and pH dynamics were performed using the VisiSens TD planar optode system (PreSens – Precision Sensing GmbH) which integrates a fluorescent imaging setup, allows for the quantification of chemical gradients with high spatial resolution (~25 µm) (Wang, 2010; Kumari and Gupta, 2017). The optode foils employed in these experiments were SF-RPSu4, 0–100% air saturation). For achieving fine-scale resolution, a specialised microscope lens equipped with an excitation light source was utilised (PreSens - TD MIC kit, field of view approximately 2.5 mm x 1.8 mm). Oxygen concentration was determined using the VisiSens TD software and false colour images created to illustrate oxygen gradients (Supplementary Figure S3B).
