# Supplementary material for: Respiratory quotients of particle-associated microbes track carbon flux attenuation in the mesopelagic Southern Ocean
Source: ISME J. 2025 Nov 20;19(1):wraf255. doi: 10.1093/ismejo/wraf255 (PMC12694406; doi:10.1093/ismejo/wraf255)
Supplement: Supplementary_Materials_wraf255 [file supplementary_materials_wraf255.docx]

**Supplementary**

**Table S1 Depth log of the deployment depth of the pit (denoted by ST) and the c-respire (denoted by R) traps.** Deployment depths varied between sites due to differences in the mixed layer depths. Respires and pit traps could not be deployed on the same array and so c-respire traps were deployed 20 m deeper at each site (based on the freeboard of the ship).

| Deployment | Deployment | Recovery_  UTC | Lat_  deployment | Lon_  deployment | Trap  Type | Mean  depth |
| --- | --- | --- | --- | --- | --- | --- |
| SOL1 | 8/12/2020 23:45 | 11/12/2020 23:45 | -47.077 | 141.394 | ST1 | 179.0 |
|  |  |  |  |  | R1 | 198.9 |
|  |  |  |  |  | ST2 | 229.0 |
|  |  |  |  |  | R2 | 248.9 |
|  |  |  |  |  | ST3 | 272.0 |
|  |  |  |  |  | R3 | 292.1 |
| SOL2 | 12/12/2020 23:15 | 15/12/2020 22:30 | -47.088 | 141.374 | ST1 | 179.0 |
|  |  |  |  |  | R1 | 198.9 |
|  |  |  |  |  | ST2 | 229.0 |
|  |  |  |  |  | R2 | 248.9 |
|  |  |  |  |  | ST3 | 275.0 |
|  |  |  |  |  | R3 | 294.9 |
| SOL3 | 16/12/2020 20:00 | 19/12/2020 22:30 | -47.082 | 141.366 | ST1 | 185.0 |
|  |  |  |  |  | R1 | 204.8 |
|  |  |  |  |  | ST2 | 232.0 |
|  |  |  |  |  | R2 | 251.7 |
|  |  |  |  |  | ST3 | 277.0 |
|  |  |  |  |  | R3 | 297.4 |
| SOL4 | 23/12/2020 23:50 | 26/12/2020 22:15 | -55.918 | 139.138 | ST1 | 189.0 |
|  |  |  |  |  | R1 | 209.2 |
|  |  |  |  |  | ST2 | 237.0 |
|  |  |  |  |  | R2 | 256.7 |
|  |  |  |  |  | ST3 | 282.0 |
|  |  |  |  |  | R3 | 302.4 |
| SOL5 | 27/12/2020 21:45 | 30/12/2020 22:00 | -55.802 | 138.623 | ST1 | 189.0 |
|  |  |  |  |  | R1 | 208.9 |
|  |  |  |  |  | ST2 | 236.0 |
|  |  |  |  |  | R2 | 255.9 |
|  |  |  |  |  | ST3 | 282.0 |
|  |  |  |  |  | R3 | 302.4 |
| SOL6 | 31/12/2020 22:30 | 3/01/2021 22:00 | -58.053 | 141.259 | ST1 | 192.0 |
|  |  |  |  |  | R1 | 212.0 |
|  |  |  |  |  | ST2 | 239.0 |
|  |  |  |  |  | R2 | 259.4 |
|  |  |  |  |  | ST3 | 285.0 |
|  |  |  |  |  | R3 | 305.2 |
| SOL7 | 4/01/2021 22:15 | 7/01/2021 22:00 | -57.981 | 141.374 | ST1 | 191.0 |
|  |  |  |  |  | R1 | 211.3 |
|  |  |  |  |  | ST2 | 239.0 |
|  |  |  |  |  | R2 | 259.4 |
|  |  |  |  |  | ST3 | 284.0 |
|  |  |  |  |  | R3 | 304.4 |

| **Table S2: ARQs were corrected for nitrification using concurrent c-respire datasets. For comparison, total nitrification demand and the associated oxygen consumption were estimated based on nitrification rates reported by Liu and Wang (2012). This calculation assumes an oxygen demand of 1.86 µmol O₂ per µmol N (equivalent to 4.23 mg O₂ per mg N).** | | | | | | | | |
| --- | --- | --- | --- | --- | --- | --- | --- | --- |
|  |  |  |  |  | **Corrected for Nitrification** | | **Total nitrification** | |
| **Site** | **Lat** | **Lon** | **Depth** | **mean ARQ*** | **NO_2_ release rate** | **mean ARQ** | 4.23 mg O_2_ per mg N | mean ARQ (1.86 nitrification) |
| **PIT** |  |  |  |  | **μmol N /L/d** | | **≈ 1.86 µmol O₂ per µmol N** |  |
| **SOTS1** | -47.08 | 141.39 | 179 | 0.7224 | 0.003 | 0.7222 | 0.0056 | 0.7220 |
|  |  |  | 229 | 0.2462 | 0.02 | 0.2460 | 0.0372 | 0.2458 |
|  |  |  | 272 | 0.3685 | 0.02 | 0.3671 | 0.0372 | 0.3658 |
| **SOTS2** | -47.09 | 141.37 | 179 | 0.7729 | 0.01 | 0.7724 | 0.0186 | 0.7720 |
|  |  |  | 229 | 0.2943 | 0.02 | 0.2940 | 0.0372 | 0.2938 |
|  |  |  | 275 | 0.4918 |  | 0.4918 |  | 0.4900 |
| **SOTS3** | -47.08 | 141.37 | 185 | 0.8315 | 0.01 | 0.8310 | 0.0186 | 0.8306 |
|  |  |  | 232 | 0.6951 | 0.01 | 0.6949 | 0.0186 | 0.6948 |
|  |  |  | 277 | 0.8481 |  | 0.8477 |  | 0.8472 |
| **PF1_1** | -55.92 | 139.14 | 189 | 0.6446 | 0.01 | 0.6444 | 0.0186 | 0.6441 |
|  |  |  | 237 | 0.3538 | 0.01 | 0.3537 | 0.0186 | 0.3536 |
|  |  |  | 282 | 0.2484 | 0.02 | 0.2483 | 0.0372 | 0.2483 |
| **PF1_2** | -55.8 | 138.62 | 189 | 0.7524 | 0.01 | 0.7520 | 0.0186 | 0.7518 |
|  |  |  | 236 | 0.5387 | 0.01 | 0.5386 | 0.0186 | 0.5385 |
|  |  |  | 282 | 0.3214 | 0.01 | 0.3213 | 0.0186 | 0.3213 |
| **PF2_1** | -58.05 | 141.26 | 192 | 0.8601 | -0.04 | 0.8604 | -0.0744 | 0.8606 |
|  |  |  | 239 | 0.6488 | -0.01 | 0.6489 | -0.0186 | 0.6490 |
|  |  |  | 285 | 0.3893 | 0.01 | 0.3892 | 0.0186 | 0.3892 |
| **PF2_2** | -57.98 | 141.37 | 191 | 0.8620 | -0.02 | 0.8623 | -0.0372 | 0.8625 |
|  |  |  | 239 | 0.4129 | 0.03 | 0.4127 | 0.0558 | 0.4126 |
|  |  |  | 284 | 0.3890 | 0.03 | 0.3889 | 0.0558 | 0.3888 |
|  |  |  |  |  | **NO_2_ release rate** | **mean ARQ** |  |  |
| **c-respire** | | |  |  | **μmol N /L/d** | |  |  |
| **SOTS1** | -47.08 | 141.39 | 199 | 0.4162 | 0.003 | 0.4162 | 0.0056 | 0.4161 |
|  |  |  | 245 | 0.2444 | 0.02 | 0.2444 | 0.0372 | 0.2442 |
|  |  |  | 292 | 0.2626 | 0.02 | 0.2626 | 0.0372 | 0.2621 |
| **SOTS2** | -47.09 | 141.37 | 203 | 0.3679 | 0.01 | 0.3679 | 0.0186 | 0.3674 |
|  |  |  | 249 | 0.1654 | 0.02 | 0.1654 | 0.0372 | 0.1653 |
|  |  |  | 295 | 0.1038 |  | 0.1038 |  | 0.1038 |
| **SOTS3** | -47.08 | 141.37 | 205 | 0.2916 | 0.01 | 0.2916 | 0.0186 | 0.2914 |
|  |  |  | 252 | 0.2117 | 0.01 | 0.2117 | 0.0186 | 0.2116 |
|  |  |  | 299 | 0.4178 |  | 0.4178 |  | 0.4178 |
| **PF1_1** | -55.92 | 139.14 | 209 | 0.1283 | 0.01 | 0.1283 | 0.0186 | 0.1283 |
|  |  |  | 256 | 0.3536 | 0.01 | 0.3536 | 0.0186 | 0.3535 |
|  |  |  | 302 | 0.2283 | 0.02 | 0.2283 | 0.0372 | 0.2282 |
| **PF1_2** | -55.8 | 138.62 | 209 | 0.1742 | 0.01 | 0.1742 | 0.0186 | 0.1742 |
|  |  |  | 256 | 0.1534 | 0.01 | 0.1534 | 0.0186 | 0.1534 |
|  |  |  | 303 | 0.5076 | 0.01 | 0.5076 | 0.0186 | 0.5074 |
| **PF2_1** | -58.05 | 141.26 | 212 | 0.0995 | -0.04 | 0.0995 | -0.0744 | 0.0996 |
|  |  |  | 259 | 0.3824 | -0.01 | 0.3824 | -0.0186 | 0.3825 |
|  |  |  | 305 | 0.5293 | 0.01 | 0.5293 | 0.0186 | 0.5291 |
| **PF2_2** | -57.98 | 141.37 | 211 | 0.1839 | -0.02 | 0.1839 | -0.0372 | 0.1841 |
|  |  |  | 258 | 0.1075 | 0.03 | 0.1075 | 0.0558 | 0.1075 |
|  |  |  | 304 | 0.2117 | 0.03 | 0.2117 | 0.0558 | 0.2115 |

**Table S3: Key characteristics of the samples acquired from pits or C-RESPIRE and subsequently incubated shipboard to measure ARQs.** “n=site” denotes the total number of site-specific samples collected across all depths. "n" is the total number of samples included in the analysis at each depth following removal of unsuitable samples. "n removed" refers to the number of samples that were excluded from the analysis due to occasional sensor issues or the presence of zooplankton within the sample. Sites are from subantarctic (SOTS1, 2, 3 deployments) and south of the Polar Front (gray - PF1; 1, 2 deployments); black - PF2, 1, 2 deployments). Temp denotes surface-mixed layer temperature that was used to set the temperature control lab temperature of both subpolar and polar incubations to assess ARQ. Mixed layer temperature was used as only one temperature control facility was available on the ship.

| **Site** | **Lat** | **Lon** | **n=site** | **Temp (^o^C)** | **Depth** | **n** | **n removed** | **ARQ mean** | **ARQ std** |
| --- | --- | --- | --- | --- | --- | --- | --- | --- | --- |
| **PIT** |  |  |  |  |  |  |  |  |  |
| **SOTS1** | -47.08 | 141.39 | 31 | 8.8 | 179 | 10 | 6 | 0.76 | 0.44 |
|  |  |  |  |  | 229 | 3 | 13 | 0.26 | 0.15 |
|  |  |  |  |  | 272 | 4 | 12 | 0.39 | 0.34 |
| **SOTS2** | -47.09 | 141.37 | 36 | 8.7 | 179 | 16 | 0 | 0.87 | 0.33 |
|  |  |  |  |  | 229 | 9 | 7 | 0.29 | 0.24 |
|  |  |  |  |  | 275 | 8 | 8 | 0.55 | 0.29 |
| **SOTS3** | -47.08 | 141.37 | 39 | 9 | 185 | 16 | 0 | 0.93 | 0.42 |
|  |  |  |  |  | 232 | 15 | 1 | 0.78 | 0.57 |
|  |  |  |  |  | 277 | 14 | 2 | 0.95 | 0.67 |
| **PF1_1** | -55.92 | 139.14 | 40 | 4 | 189 | 16 | 0 | 0.69 | 0.31 |
|  |  |  |  |  | 237 | 15 | 1 | 0.38 | 0.12 |
|  |  |  |  |  | 282 | 9 | 7 | 0.24 | 0.08 |
| **PF1_2** | -55.8 | 138.62 | 36 | 6.2 | 189 | 16 | 0 | 0.85 | 0.42 |
|  |  |  |  |  | 236 | 10 | 6 | 0.56 | 0.56 |
|  |  |  |  |  | 282 | 16 | 0 | 0.35 | 0.13 |
| **PF2_1** | -58.05 | 141.26 | 43 | 5.7 | 192 | 16 | 0 | 0.86 | 0.50 |
|  |  |  |  |  | 239 | 13 | 3 | 0.65 | 0.38 |
|  |  |  |  |  | 285 | 13 | 3 | 0.39 | 0.20 |
| **PF2_2** | -57.98 | 141.37 | 41 | 4.9 | 191 | 16 | 0 | 0.90 | 0.43 |
|  |  |  |  |  | 239 | 13 | 3 | 0.43 | 0.22 |
|  |  |  |  |  | 284 | 9 | 7 | 0.37 | 0.15 |
| **C-RESPIRE** | | | |  |  |  |  |  |  |
| **SOTS1** | -47.08 | 141.39 | 16 | 8.8 | 199 | 10 | 0 | 0.44 | 0.20 |
|  |  |  |  |  | 245 | 9 | 1 | 0.26 | 0.30 |
|  |  |  |  |  | 292 | 9 | 1 | 0.28 | 0.26 |
| **SOTS2** | -47.09 | 141.37 | 12 | 8.7 | 203 | 4 | 0 | 0.41 | 0.23 |
|  |  |  |  |  | 249 | 4 | 0 | 0.19 | 0.07 |
|  |  |  |  |  | 295 | 2 | 2 | 0.12 | 0.05 |
| **SOTS3** | -47.08 | 141.37 | 12 | 9 | 205 | 4 | 0 | 0.33 | 0.35 |
|  |  |  |  |  | 252 | 4 | 0 | 0.24 | 0.25 |
|  |  |  |  |  | 299 | 4 | 0 | 0.47 | 0.49 |
| **PF1_1** | -55.92 | 139.14 | 12 | 4 | 209 | 3 | 1 | 0.15 | 0.11 |
|  |  |  |  |  | 256 | 4 | 0 | 0.38 | 0.17 |
|  |  |  |  |  | 302 | 4 | 0 | 0.25 | 0.15 |
| **PF1_2** | -55.8 | 138.62 | 12 | 6.2 | 209 | 4 | 0 | 0.20 | 0.10 |
|  |  |  |  |  | 256 | 4 | 0 | 0.22 | 0.06 |
|  |  |  |  |  | 303 | 4 | 0 | 0.58 | 0.19 |
| **PF2_1** | -58.05 | 141.26 | 9 | 5.7 | 212 | 3 | 1 | 0.10 | 0.05 |
|  |  |  |  |  | 259 | 4 | 0 | 0.38 | 0.20 |
|  |  |  |  |  | 305 | 2 | 2 | 0.53 | 0.48 |
| **PF2_2** | -57.98 | 141.37 | 10 | 4.9 | 211 | 4 | 0 | 0.19 | 0.06 |
|  |  |  |  |  | 258 | 3 | 1 | 0.11 | 0.07 |
|  |  |  |  |  | 304 | 3 | 1 | 0.22 | 0.13 |

**Table S4*:* Site-specific characteristics of Southern Ocean sample sites.** ML is the mixed layer depth in meters. NPP is the net primary productivity (mg C m^-2^ d^-1^) integrated to the base of the euphotic zone. T_eff_ refers to the transfer efficiency of POC over a 100 m depth stratum in the upper mesopelagic. *b*-value is the POC flux attenuation coefficient, calculated using the Martin et al. (1987) equation. ARQ refers to the mean ARQ at each depth stratum and site (<200 m, >230 m).

| **Site** | **ML depth (m)** | **NPP** | **Teff (%)** |  | ***b-*value** | **ARQ <200 m** | **ARQ > 230 m** | **Particle typology** |
| --- | --- | --- | --- | --- | --- | --- | --- | --- |
|  |  | **(mg C m^-2^ d^-1^)** |  |  |  |  |  |  |
| **SOTS** | 50 | 2263-3112 | 75 |  | 0.64 ± 0.31 | 0.86 | 0.64 | Small dense aggregates and fecal pellet |
| **PF1** | 65 | 469-642 | 75 |  | 0.69 ± 0.41 | 0.77 | 0.42 | Very large and fluffy fecal pellets |
| **PF2** | 75 | 1222-1785 | 48 |  | 0.7± 0.36 | 0.87 | 0.46 | Phytodetrital and diatom cells |

**Table S5 Initial and final dissolved oxygen data from the c-respire incubations during the SOLACE voyage.**

| Deployment_ID | Depth_m | Lat | Lon | initial_O_2__ μM | final_O_2__ μM | O_2__consumed_ μM |
| --- | --- | --- | --- | --- | --- | --- |
| RES_SOL1 | 198.87 | -47.08 | 141.39 | 258.20 | 245.80 | 12.40 |
| RES_SOL1 | 245.48 | -47.08 | 141.39 | 258.30 | 241.80 | 16.50 |
| RES_SOL1 | 292.08 | -47.08 | 141.39 | 260.81 | 244.50 | 16.31 |
| RES_SOL2 | 202.90 | -47.09 | 141.37 | 263.34 | 259.00 | 4.34 |
| RES_SOL2 | 248.92 | -47.09 | 141.37 | 235.18 | 192.10 | 43.08 |
| RES_SOL2 | 294.94 | -47.09 | 141.37 | 249.52 | 208.40 | 41.12 |
| RES_SOL3 | 204.80 | -47.08 | 141.37 | 247.94 | 228.70 | 19.24 |
| RES_SOL3 | 251.73 | -47.08 | 141.37 | 262.78 | 250.20 | 12.58 |
| RES_SOL3 | 298.66 | -47.08 | 141.37 | 260.18 | 244.40 | 15.78 |
| RES_SOL4 | 209.17 | -55.92 | 139.14 | 264.90 | 259.30 | 5.60 |
| RES_SOL4 | 255.78 | -55.92 | 139.14 | 246.89 | 238.40 | 8.49 |
| RES_SOL4 | 302.38 | -55.92 | 139.14 | 231.58 | 227.40 | 4.18 |
| RES_SOL5 | 208.89 | -55.80 | 138.62 | 252.79 | 246.50 | 6.29 |
| RES_SOL5 | 255.93 | -55.80 | 138.62 | 249.88 | 244.50 | 5.38 |
| RES_SOL5 | 302.97 | -55.80 | 138.62 | 233.00 | 226.70 | 6.30 |
| RES_SOL6 | 211.98 | -58.05 | 141.26 | 314.90 | 311.30 | 3.60 |
| RES_SOL6 | 258.61 | -58.05 | 141.26 | 293.17 | 287.50 | 5.67 |
| RES_SOL6 | 305.23 | -58.05 | 141.26 | 252.02 | 248.20 | 3.82 |
| RES_SOL7 | 211.29 | -57.98 | 141.37 | 305.23 | 290.80 | 14.43 |
| RES_SOL7 | 257.85 | -57.98 | 141.37 | 277.21 | 272.40 | 4.81 |
| RES_SOL7 | 304.42 | -57.98 | 141.37 | 239.34 | 235.70 | 3.64 |

**Table S6 Particle size (microns), sinking speed and penetration depth derived from a UVP6 particle imaging system on a profiling robotic float (BGC-ARGO) deployed at SOTS on a four-year mission during the SOLACE voyage**. *denotes that particles <406 microns did not penetrate the water column to the depth of the shallowest trap (data from Lacour et al., submitted).

| **Size class label** | **ESD** | **Sinking speed** | **Penetration depth** |
| --- | --- | --- | --- |
| LPM_102 | 0.11 | 2.97 | 99 |
| LPM_128 | 0.14 | 3.19 | 99 |
| LPM_161 | 0.18 | 4.29 | 109 |
| LPM_203 | 0.23 | 4.66 | 113 |
| LPM_256 | 0.29 | 5.29 | 119 |
| LPM_323 | 0.36 | 6.41 | 123 |
| LPM_406 | 0.46 | 8.7 | 145* |
| LPM_512 | 0.58 | 13.23 | 181 |
|  |  |  |  |
| LPM_645 | 0.73 | 17.17 | 236 |
| LPM_813 | 0.92 | 19.26 | 233 |
| LPM_1020 | 1.16 | 33.52 | 297 |
| LPM_1290 | 1.46 | 47.61 | 377 |
| LPM_1630 | 1.84 | 76.78 | 642 |
| LPM_2050 | 2.31 | 136.53 | 842 |

**Supplementary Figure captions**

**Figure S1:** Functioning of the c-respire particle interceptor/ incubator redrawn from Bressac et al. (2024). The operation of the c-respire particle interceptor/incubator is presented as a conceptual time-series of particle interception (a-c) and rapid transfer of particles (rotating indented solid sphere every 10 min illustrated by the green arrow) into an inner chamber housing an oxygen optode that records dissolved oxygen (DO) every 5 min (denoted in each panel by change from yellow to gray background). Prior to deployment, the inner chamber is filled with 0.2 micron filtered seawater collected at the deployment depth. Panels d-f illustrate the incubation phase after the periodically rotating sphere is pre-programmed to halt and the optode tracks the decrease in DO over time. POC remaining at the end of the multi-day incubation is recovered (then analysed) from the inner chamber after first siphoning off all water and particles overlying the sphere within the c-respire. The exclusion of zooplankton by the solid sphere results in the intercepted particles only being degraded by microbes during the incubation.

**Figure S2:** Schematic of the two trap systems (pit and c-respire) used to intercept the particle assemblages**. A)** Particles settled into unpreserved seawater within particle interceptor traps (pit), then incubated shipboard for ARQ determination on ‘fresh’ particles. Particles will have been transformed during sinking and to some extent when intercepted (between <72 h and > 0 h over the 3-day collection period). **B)** c-respire - particles intercepted (< 36 h > 0 h before in situ incubation when the indented rotating sphere is programmed to halt denoting the start of the in situ incubation) and then incubated in situ (36 hours, during which time additional particles settle onto the upper side of the static indented sphere (denoted by the upper part of the sphere being coated), After recovery of c-respire, particles from the inner chamber below the indented sphere were collected and further incubated shipboard for ARQ determination of “pre-degraded” particles. These particles have been subject to 36 h of microbial degradation during their incubation and should have lower ARQ prior to further incubation shipboard.

**Figure S3**: Illustration of the PreSens imaging system used to determine particle ARQs. A) Particles were collected and placed in sealed vials (4 mL, Merck, Z256064) and O_2_, and CO_2_ concentrations determined over a 12-h period in a constant temperature lab using a PreSens O_2_ (SF-RPSu4) and a CO_2_ (SF-CD2R) sensor. We could only measure CO_2_ and therefore cannot consider any subsequent interplay with other components of carbonate chemistry. Glass vials containing particles were placed in individual wells of a 24-well flat-bottom plate (Corning CLS3527). Each plate contained 16 samples from unpreserved pits, four from c-respire, and four negative control vials (filtered seawater collected at the site/depth). The sample vials were incubated for 12 h at ambient seawater temperature (Table S1), with readings taken every 6 h using the VisiSens TD (PreSens, Germany). Subsequently, all samples were processed for preservation using 0.5% glutaraldehyde for further microscopic analysis. If zooplankton were present within a sample, the samples were removed from the subsequent analysis to ensure estimation of microbially mediated particle degradation. B) Illustration of the placement of the krill fecal pellets, in lab experiments, in a transparent, 3D-printed flow chamber (3 mm x 20 mm) equipped with oxygen and pH optodes. Oxygen and pH dynamics were performed using the VisiSens TD planar optode system (PreSens – Precision Sensing GmbH) which integrates a fluorescent imaging setup, allows for the quantification of chemical gradients with high spatial resolution (~25 µm). Oxygen concentration was determined using the VisiSens TD software and false colour images created to illustrate oxygen gradients.

**Figure S4:** Vertical profiles of the ratio of the POC and chlorophyll a fluxes intercepted at three depths and 3 sites (color-coded as for Fig. 2A). Error bars denote the standard deviation of the mean.

**Figure S5:** A) Illustrative example of particle types collected in polyacrylamide gel traps at the same depth but at different sites in the present study. Particles from SOTS site contained generally small, compact particles with a high proportion of fecal pellets. Whereas particles from the two polar front sites (only PF1 is shown, large and less dense salp fecal pellets, c.f. PF which also had large, porous aggregates, some of algal origin. B) a subantarctic zooplankton fecal pellet under epifluorescence microscopy reveals traces of red auto-fluorescing chlorophyll that likely comprised much of the observed downward chlorophyll fluxes in Figure 2.
